# Oral and Laryngeal Articulation Control of Voicing in Children with and without Speech Sound Disorders

**DOI:** 10.3390/children9050649

**Published:** 2022-05-01

**Authors:** Haydée Fiszbein Wertzner, Luciana Pagan Neves, Luis M. T. Jesus

**Affiliations:** 1 Department of Physical Therapy, Speech-Language Pathology and Audiology and Occupational Therapy of the School of Medicine at the University of São Paulo (HC/FMUSP), University of São Paulo, São Paulo 01246903, Brazil; hfwertzn@usp.br (H.F.W.); lucianapagan@hotmail.com (L.P.N.); 2 School of Health Sciences (ESSUA), Institute of Electronics and Informatics Engineering of Aveiro (IEETA), University of Aveiro, 3810-193 Aveiro, Portugal

**Keywords:** speech sound disorders, children, speech production, speech, language and hearing assessment

## Abstract

Voicing contrast is hard to master during speech motor development, and the phonological process of consonant devoicing is very frequent in children with Speech Sound Disorders (SSD). Therefore, the aim of this study was to characterise the oral and laryngeal articulation control strategies used by children with and without SSD as a function of place of articulation. The articulation rate and relative oral airflow amplitude (*flow*) were used to analyse how children controlled oral articulation; fundamental frequency (*f_o_*), open quotient (OQ), and a classification of voicing were used to explore laryngeal behaviour. Data from detailed speech and language assessments, oral airflow and electroglottography signals were collected from 13 children with SSD and 17 children without SSD, aged 5; 0 to 7; 8, using picture naming tasks. Articulation rate and *flow* in children with and without SSD were not significantly different, but a statistically reliable effect of place on *flow* was found. Children with and without SSD used different relative *f_o_* (which captures changes in *f_o_* during the consonant-vowel transition) and OQ values, and place of articulation had an effect on the strength of voicing. All children used very similar oral articulation control of voicing, but children with SSD used less efficient laryngeal articulation strategies (higher subglottal damping and more air from the lungs expelled in each glottal cycle) than children without SSD.

## 1. Introduction

Speech sound disorders (SSD) is an umbrella term used in cases of children presenting any difficulty or combination of difficulties with perception, motor production, or phonological representation of speech sounds and is one of the most prevalent disorders in children under six years of age. Children with SSD may present sound omission, substitution, addition and/or distortion. Many children with SSD present phonological processes that typically developing children use to simplify speech while their speech and language are developing [1,2,3].

One of the most frequent phonological impairments in Brazilian Portuguese-speaking children with SSD is the phonological process of fricative and stop devoicing [4,5]. Substitution processes that lead to changes in voicing have been identified as one of the most common developmental processes [1,6,7]: Children replace a voiceless sound by a voiced sound (phonological process of voicing) or replace a voiced sound by a voiceless sound (phonological process of devoicing). These phonological processes include devoicing of initial consonants [6] or voicing unvoiced sounds, variable realisations of voiced-voiceless cognates and prevocalic voicing [1].

The oral and laryngeal articulation control characteristics, vocal fold open quotient (OQ), fundamental frequency (*f_o_*) and amplitude of the oral airflow of fricatives and stops could contribute important evidence to the understanding of the strategies that lead to devoicing in children with SSD. Some Brazilian acoustic studies have previously documented the production of phonologically voiced sounds without vocal fold vibration [8,9,10] and studies of Portuguese children have also shown a frequent use of the phonological process of devoicing [6]. Jesus et al. [6] analysed the percentage of fricative devoicing in both children with SSD and typically developing children with the same chronological age. The authors [6] observed that children with SSD had a significantly higher percentage of occurrence of fricative devoicing when compared with typically developing children. Both Brazilian and Portuguese studies have also pointed out that most children with SSD present fricative devoicing until they are five years old [4,6,7].

Another factor that has been shown to condition voicing in fricatives is the place of articulation. Studies that have classified voicing using just the acoustic signal [11]—both the acoustic and electroglottography (EGG) signals [12] and only the relative oral airflow signal [13]—have all shown that Portuguese speakers’ percentage of devoicing was higher for the more posterior places of articulation.

### 1.1. Voicing Consonants

Several factors may interfere with consonant voicing in children [14], so, despite numerous recent advances in techniques and equipment available for speech analysis, the control mechanisms of vocal fold vibration are not yet fully understood. According to the myoelastic aerodynamic theory of voice production [15], phonation starts from complete adduction of the vocal folds to close the glottis, which allows for a build-up of the subglottal pressure until it is sufficiently high enough to push them apart [16]. The production of voiced sounds requires both control of the glottal opening and the passage of sufficient air through the glottis at an adequate velocity to sustain vibration. An increase in intraoral air pressure (that usually occurs during fricative and stop consonant production) decreases the pressure differential across the glottis which does not facilitate vocal fold vibration [12].

Both cognitive-linguistic and motor aspects of speech production should be analysed in order to study voicing of children’s speech sounds. Boucher and Lamontagne [17] have shown that there are two different modes of voicing control that operate at different rates of speech. Thus, with low speech rate, control of voicing occurs via glottal movement and/or airflow, which may hinder or interrupt vocal fold vibration. With a high speech rate, there is a change in the velocity of the airflow, pressure release movements that can lead to a rapid increase in the amplitude of the oral airflow which is responsible for the onset of vocal fold vibration.

### 1.2. Aerodynamic Measures

The specific way children learn to combine laryngeal and oral actions when voicing fricatives has, however, been seldom studied [18]. We therefore propose, in the present study, novel acoustic, aerodynamic and electroglottography-based methods [13] that can be used to analyse children’s voicing of fricatives. The use of aerodynamic measures can be very helpful in this context since such measures are more directly related to speech production mechanisms and may offer new insights into the maintenance and cessation of voicing in different phonological contexts [19]. Aerodynamic conditions, including amplitude of airflow, transglottal pressure and intraoral pressure, have frequently been shown to differ for consonant voicing cognates [19,20]. Studying how the aerodynamics of speech sounds change in different conditions may also contribute to a better understanding of the dynamic characteristics of different phonological systems [21]. The analysis of the *f_o_* and OQ of vocal fold oscillation, and of the relative amplitude of the oral airflow have been previously used [13,19] as the basis of such studies.

The *f_o_* indicates changes of the mechanical properties of the vocal folds and changes in the transglottal air pressure (increased longitudinal tension of the vocal folds and laryngeal lowering). Lower amplitudes of the oral airflow and a decrease in *f_o_* values are related to weaker voicing [13,19]. In general, voiced fricatives tend to have lower *f_o_* than adjacent vowels due to laryngeal lowering with a greater longitudinal tension of the vocal folds [13,22]. The values of *f_o_* reflect voicing strategies in sound production that combine laryngeal lowering with the abduction of the vocal folds and the transglottal pressure, both of which are required to maintain oscillation. Therefore, a relative amplitude oral airflow measure that relates the characteristics of the voiced fricatives to those of the vowels that are produced before or after it, can reveal the strategies used by children to maintain voicing. A relative amplitude higher than 70% has been previously proposed [13] as a classification criterion for weak voicing as compared to the strong voicing typically observed in adjacent vowels.

### 1.3. Electroglottographic Measures

Electroglottography (EGG) offers new insights to identify difficulties that children find in producing voicing in consonants. The values of the open and closed quotients of the vocal folds can be estimated from the EGG signal [23]. The open quotient (OQ) refers to the proportion of time (relative to the fundamental period) during which the glottis remains adducted [24,25] and is a measure used to determine the efficiency of the vocal fold closure [26]. Associated with aerodynamic measurements, it may reveal novel strategies for voicing production in children.

### 1.4. Articulation Rate

The Articulatory Rate (AR) reflects the maturity of the speech motor system, but cannot be used as an isolated motor measure as it incorporates aspects related to cognitive-linguistic processing of information [27,28], including an increased load in phonological and syntactic processing at the age of five years [29].

The AR can be used as a complementary measure for the description of SSD since it is related to the neuromuscular capacities and to the maturity of the motor speech system and can also be correlated to measures based on oral airflow and EGG signals that reflect voicing control.

The AR is measured as the number of syllables and/or phones produced per second and may be associated with two factors [30,31,32,33]: neuromuscular capacities and/or sociolinguistic aspects. Previous work [30,34,35] has shown that AR increases with age in typical speech and language development and also that both children and adolescents show lower AR than adults [36,37].

The effect of the extension of the sentence on AR measures has also been studied. Children with typical speech and language development have higher AR in long sentences than in short ones [33,37]. This phenomenon may be related to the strategies used to control coarticulation and thus generate an increase in the number of phones per second that are produced during speech. Thus, in structured situations, in which it is possible to control the occurrence of pauses (as in the repetition of sentences), the influence of language formulation on speech rate is minimised, better reflecting the performance of motor mechanisms of speech production [31].

Some studies have shown that children with SSD have lower AR than those with typical development. Therefore, the analysis of this measure could help in the intervention with children with SSD that reveal difficulties controlling speech rate [30,31,33,38].

## 2. Motivation and Aims of this Study

Since the phonological process of devoicing is one of the most frequent in children with SSD, functional evaluation of voiced sounds production and the articulatory rate (phones/s) can provide important evidence for an effective speech and language intervention.

Considering that the aerodynamic control of fricative production and maintenance of voicing in fricatives depends on several factors, the aim of this study was to analyse the maturity in speech voicing control in children with and without SSD, based on aerodynamic and EGG -based measures, for three places of articulation.

## 3. Method

The Research Ethics Committee of the Faculty of Medicine of the University of São Paulo in Brazil approved the Research Protocol No. 036/14, described below. All the parents or guardians signed a consent form and all the children agreed to participate in the study.

### 3.1. Children

A total of 55 children, from a waiting list at the Research Laboratory in Phonology within the department of Physical Therapy, Speech-Language Pathology and Audiology and Occupational Therapy of the School of Medicine at the University of São Paulo (HC/FMUSP) were recruited and evaluated. Four children presenting speech and language disorders (other than SSD) were not included in the study and were referred to an external Speech and Language Pathologist. Four children with SSD did not cooperate in the acquisition of the aerodynamic and EGG signals. In addition, for 17 of the 47 included children, sample losses (detailed below) for a few sounds occurred: It was not possible to acquire the acoustic, oral airflow or EGG signals, or to determine the articulatory rate. This was due to equipment malfunction at the time of data acquisition, which did not allow adequate data recording.

Inclusion criteria for children with SSD were: age between 5; 0 and 7; 11 years (60 to 95 months); presence of phonological processes not expected for a particular age when assessed with the phonology test for Brazilian Portuguese-speakers, which is part of the ABFW Children’s Language Test [39]; age-appropriate performance in the other language skills [40]; audiological evaluation within the limits of normality (thresholds below 20 dB in frequencies 500, 1000, 2000, and 4000 Hz); non-verbal IQ within the limits of normality (WISC-III Wechsler Intelligence Scale for Children 3rd Edition); Brazilian Portuguese as the children’s first language, as well as their parents.

The control group (CG) consisted of children with typical speech and language development for their age group, recruited from a Primary School in the city of São Paulo, Brazil and from a group of voluntary participants. The inclusion criteria were Age between 5; 0 and 7; 11 years (60 to 95 months), no communication disorders as reported by parents or guardians and then assessed by the phonology tests [39] of the ABFW Children’s Language test, absence of auditory complaints, audiological evaluation within limits of normality (thresholds below 20 dB in frequencies 500, 1000, 2000, and 4000 Hz); Brazilian Portuguese monolingual children and parents.

### 3.2. Materials Used for Data Collection

Materials used for data collection included those from the ABFW Children’s Language test (phonology, vocabulary, fluency and pragmatics) [40], the articulation rate test, and protocols for registering data and signal analysis.

The EGG signal generated by a Glottal Enterprises Model EG2-PCX2 processor, the oral airflow signal captured by a S/T-MC1 oro-nasal two-chamber circumferentially vented (CV) child mask (Glottal Enterprises, Syracuse, NY, USA) and a PT-2E pressure transducer (Glottal Enterprises, Syracuse, NY, USA) for measuring the airflow at the mouth, were recorded with a MS 110 electronics unit (Glottal Enterprises, Syracuse, NY, USA), connected via an audio interface (iMic, Griffin Technology, Nashville, TN, USA) to a laptop computer running Waveview Pro Version 4.5 (16 bits, 44.1 kHz sampling frequency).

The phonology and AR tests were audio-recorded using a Zoom H4N audio recorder and video-recorded with a Sony HDR-CX220 camcorder (Tokyo, Japan).

All the oral airflow and EGG signals were segmented, annotated and transcribed using Praat Version 6.0.05 with Sennheiser HD650 and AKG N60 headphones (Wedemark, Germany).

### 3.3. Phonological and Articulation Rate Assessment

The assessments based on the picture naming task that is part of the phonology test from the ABFW Children’s Language Test [39] were audio and video recorded for posterior analysis. These tests were phonetically transcribed, and the phonological processes classified by two expert speech and language pathologists (SLP). Phonetic transcriptions of all children were made by two SLP (experts in SSD) and agreement between the transcriptions was ≥85%. In the present paper, the occurrence of the phonological process of fricative devoicing, total occurrence of phonological processes, number of different types of phonological processes, Percentage of Consonants Correct–Revised (PCC-R) [41] including substitutions and omissions as errors, and the Process Density Index (PDI) [42] were analysed.

The AR test was based on the production of two sentences: A short sentence (ARSS) with 12 phones <o cachorro fugiu>/<the dog ran away> and a long sentence (ARLS) with 22 phones <Maria tem uma bola vermelha>/<Maria has a red ball>. The sentences were recorded by a female adult Brazilian Portuguese-speaker. Each child was positioned facing a pair of speakers and a microphone positioned 15 cm away from the mouth. After listening to the previously recorded audio file, the children repeated each sentence three times.

The analysis of the sentence duration and AR was performed in Praat Version 6.0.05. The number of phones per second was calculated without pauses exceeding 0.15 s, as for example in a deep breath [29,30,31,36].

### 3.4. Aerodynamic, EGG and Acoustic Data Acquisition: Stimuli and Procedures

A list of words with fricatives /v, z, ʒ/ were selected (see Table 1), based on the following criteria: Target consonant in initial position of the word, target consonant followed by a high vowel and a low vowel; nouns that could be graphically represented; nouns easily recognisable by children. Visual stimuli were developed by a graphics designer and shown to five children (that did not participate in the study) to analyse their imageability. Some figures needed adjustments to be easily recognised by all children.

The visual stimuli were presented in Windows Media Player running on a laptop using a random sequence and equal time intervals between them. Four repetitions of each word were recorded, totalling 24 stimuli per participant. The equipment was calibrated before recording the oral airflow and EGG signals, and then 6 different figures were presented. A short training session was also carried out for the placement of the CV mask which was adjusted to each child for the collection of the oral airflow signal. The EEG and oral airflow signals were collected synchronously. 

### 3.5. Aerodynamic and EGG Measures

The amplitude of the oral airflow and the *f_o_* values of the consonant and the following vowel were estimated using the method proposed by Pinho et al. [13]. We then calculated the relative oral airflow and *f_o_* measures, also as in [13]: Relative measure = [(absolute measure of vowel-absolute measure of fricative)/absolute measure of vowel] × 100. Only relative oral airflow and *f_o_* values were used as outcome measures because, from a phonetic (speech production) point of view, the relative measures (from the consonant to the following vowel) are more adequate as they help to understand sound source control strategies and laryngeal behaviour when two sound sources (voiced fricatives) and one sound source (vowels) are used to produce speech.

The percentage of weak voicing [13] was also calculated. The definition of weak voicing is also based on the relative amplitude of the oral airflow, which relates production characteristics of the voiced fricative to the vowel produced after the fricative.

The *f_o_* was estimated using the Praat Version 6.0.05 autocorrelation method with the following parameters: 0 s timestep; 75Hz pitch floor; 600 Hz pitch ceiling. This is an extraction method originally designed for use with the acoustic signal which has been shown recently [13] to be a convenient method to estimate, with great precision and robustness (against ambient noise), *f_o_* from oral airflow signals. We previously [13] adapted a method that was designed for acoustic signals that are susceptible to “interference” from ambient noise to a signal that is much more “insulated” from these interferences: An oral airflow signal that is captured very close to the lips, inside a mask, that low-pass filters external noise.

The OQ was estimated from the EGG signal to analyse specific characteristics related to vocal fold vibration [8,13]. An increase in OQ is related to a less efficient voice, but in voiced fricatives it is not always possible to calculate the OQ since the EGG signal may be weak, unstable and aperiodic.

For the analysis of the three parameters (amplitude of the oral airflow, *f_o_* and OQ) data was segmented into 24 .wav files per subject. Then, all .wav files were imported into Praat Version 6.0.05, so that the fricative and the following vowel were phonetically annotated following the criteria established by Pinho et al. [13]. The analysis stage involved six Praat and Matlab 8.5.0.197613 (R2015a) scripts which extracted automatically all the measurements. It is important to note that the scripts were exactly the same as those used by Pinho et al. [13], which will allow future comparisons with their results.

### 3.6. Statistical Analysis

The boxplots presented in this paper were generated using R version 4.1.2 running in RStudio Version 1.4.1717 and include a central mark that indicates the median, the bottom and top edges of the box indicate the 25th and 75th percentiles, respectively. The whiskers extend to the first quartile (Q1) − 1.5 × Interquartile Range (IQR) and third quartile (Q3) + 1.5 × IQR [43]. The boxplots were combined with univariate scatterplots (strip plots) using the beeswarm 0.4.0 package. The datapoints are shifted horizontally to avoid superposition.

Linear regression models of each aerodynamic and EGG -based measure (relative oral airflow, relative *f_o_* and OQ) were developed with R version 4.1.2 using *group* (CG and SSD) and fricative *place* of articulation (/v/ labiodental; /z/ alveolar; /ʒ/ postalveolar) as categorical predictors. All linear regression models satisfied the normality assumption (i.e., its residuals were approximately normally distributed) and the constant variance assumption (homoscedasticity), was assessed by the following visual diagnostics plots [43]: Histogram of residuals; Q-Q plots of residuals; residuals plot.

Logistic regressions were used to model the two-alternative (strong-voiced or weak-voiced) classifications of voicing based on the relative oral airflow as a function of *place* of articulation, assuming a binomial distribution of the data-generation process [43].

## 4. Results

### 4.1. Outcomes of an Initial Speech and Language Assessment

The outcomes of an initial speech and language assessment, presented in Table 2 and Table 3, for all children that participated in the study, included the: Total number of occurrences of the phonological process of fricative devoicing (PPFD); total number of phonological processes (PPTN); total number of different phonological processes (PPDN); PCC-R; PDI; all calculated from results of a standardised picture naming task from ABFW Children’s Language Test [40].

Mean values of ARSS, ARLS, relative oral airflow (*M_flow_*), relative *f_o_* (*M_fo_*) and OQ (*M_OQ_*) are also shown for each child in Table 2 and Table 3: ARSS and ARLS were averaged across three repetitions of the sentences; *M_flow_*, *M_fo_* and *M_OQ_* are the means of all available values for each child. The relative number of fricative devoicing (RNFD) was calculated using the following formula (considering that the total number of voiced fricatives in the picture naming task was nine): RNFD = 100 × PPFD/9. The percentage of weak-voiced fricatives (RNWV) produced by each child (for all places of articulations) was computed based on the formula: 100 × total number of weak-voiced fricatives produced by the child/total number of fricatives produced by the child. The full database is available from the Supplementary Materials, WertznerPaganJesus2022_data.xlsx.

**Figure 1:**
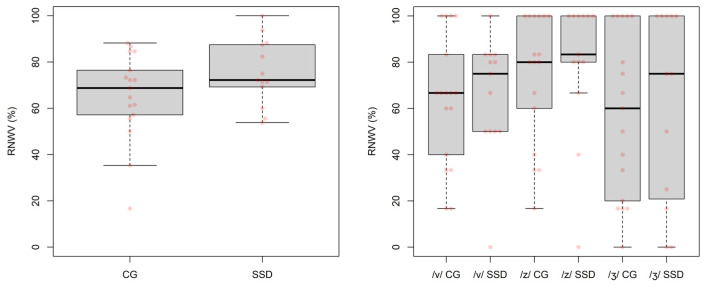
The percentage of weak-voiced fricatives (RNWV) produced by each child for all places of articulations (**left**), each place of articulation (**right**) and group (CG and SSD).

The percentage of weak-voiced fricatives (RNWV), shown in Figure 1 (left), was analysed using a regression model with RNWV as outcome variable and *group* as a binary (CG or SSD) categorical predictor, showing a positive change (*slope*) from the CG to the SSD *group* that was not significant (*slope* = 10.20, *SE* = 6.30, *p* = 0.117).

**Figure 2:**
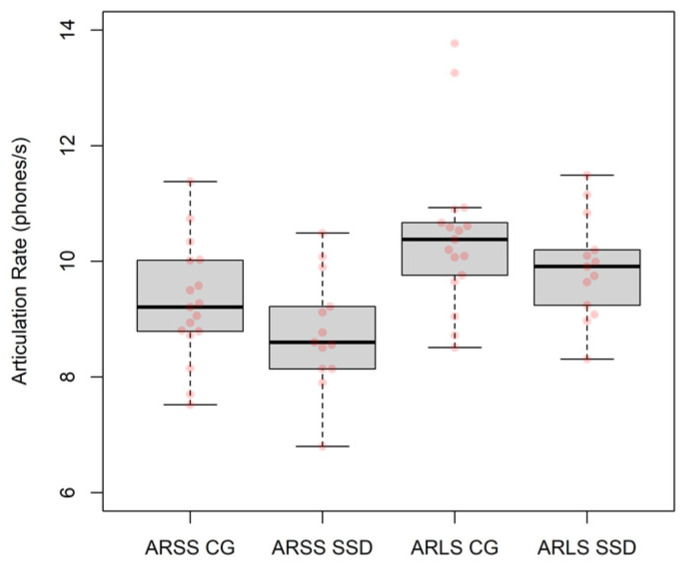
Articulation rate measures (ARSS and ARLS).

Figure 2 shows the articulation rate measures (ARSS and ARLS) for the two groups (CG and SSD). Two regression models (one for each measure) with the articulation rate as outcome variable and *group* as a two-factor categorical predictor were used to analyse the data, showing negative changes from the CG to the SSD *group* that were not significant (ARSS: *slope* = −0.49, *SE* = 0.37, *p* = 0.197; ARLS: *slope* = −0.55, *SE* = 0.215, *p* = 0.215).

The mean values of the relative oral airflow (*M_flow_*), *f_o_* (*M_fo_*) and OQ (*M_OQ_*) for each participant were used as outcome variables of three regression models (one for each measure) with *group* as a two-factor categorical predictor that revealed positive changes from the CG to the SSD *group* that were not significant for *M_flow_* (*slope* = 2.13, *SE* = 4.14, *p* = 0.610) and *M_OQ_* (*slope* = 8.55, *SE* = 5.23, *p* = 0.113), and negative changes from the CG to the SSD *group* that were significant for *M_fo_* (*slope* = −7.2, *SE* = 3.13, *p* = 0.029).

### 4.2. Aerodynamic and EGG-Based Measures

**Figure 3:**
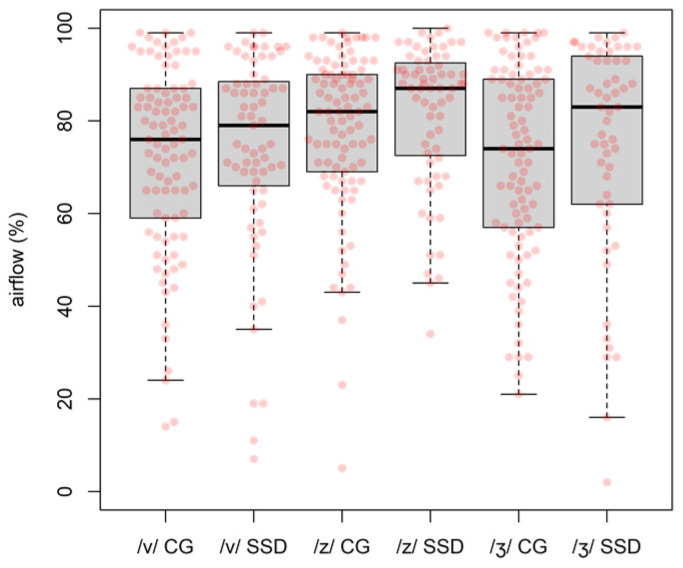
Relative oral airflow data.

Average values of the relative oral airflow amplitude measurements (see Figure 3) for children in the SSD group (*M*_/v/ SSD_ = 73.9%, *SD*_/v/ SSD_ = 21.6%, n = 67; *M*_/z/ SSD_ = 81.5%, *SD*_/z/ SSD_ = 15.6%, n = 67; *M*_/ʒ/ SSD_ = 74.8%, *SD*_/ʒ/ SSD_ = 23.9%; n = 54) were higher than those of children in the CG group (*M*_/v/ CG_ = 72.2%, *SD*_/v/ CG_ = 20.2%, n = 90; *M*_/z/ CG_ = 77.9%, *SD*_/z/ CG_ = 17.6%, n = 94; *M*_/ʒ/ CG_ = 71.5%, *SD*_/ʒ/ CG_ = 20.9%, n = 91), for each place of articulation: /v/—labiodental; /z/—alveolar; /ʒ/—postalveolar.

However, results of a regression model with relative oral airflow as outcome variable (*flow*) and *group* as a binary (CG or SSD) categorical predictor, showed a positive change (*slope*) from the CG to the SSD *group* that was not significant (*slope* = 2.97, *standard error* (*SE*) = 1.90, *p* = 0.12). A second regression model with *flow* as outcome variable and *place* as a three factor (labiodental, alveolar and postalveolar) categorical predictor revealed a statistically reliable effect of the *place* of articulation on the relative oral airflow values (*F*(2, 460) = 5.78, *p* < 0.001; omnibus test).

**Figure 4:**
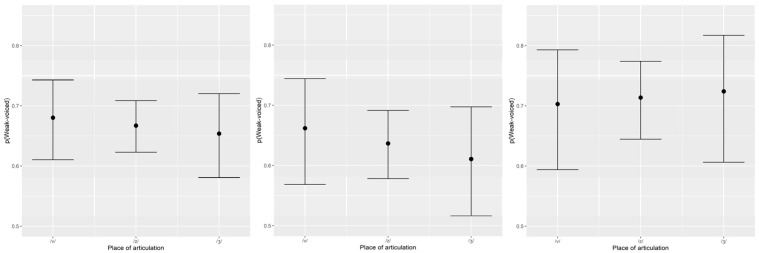
Predicted probability of observing weak-voiced fricatives for all observations (**left**), children without SSD (**middle**) and children with SSD (**right**), as a function of place of articulation.

The relative oral airflow measure was used to classify all productions of fricatives into two categories (weak-voiced–when *flow* ≥ 70.00%; strong-voiced–when *flow* < 70.00%), and three (one for all the fricatives and one for each of the groups of children) logistic regressions were used to model the two-alternative (strong-voiced or weak-voiced) classifications of voicing as a function of *place* of articulation. The plots shown in Figure 4, depicting the 95% confidence intervals around each fitted value [43], revealed that the probabilities that were predicted by the first (all fricatives–left of Figure 4) and second (CG fricatives–middle of Figure 4) models decreased as the place of articulation moved backward, and increased for the most posterior places of articulation according to the logistic regression model that was used to fit the SSD group data (right of Figure 4).

**Figure 5:**
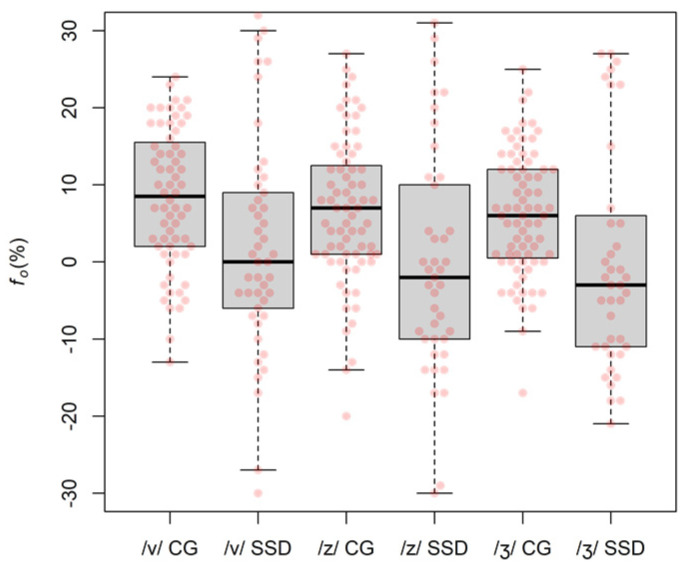
Relative *f_o_* data. Negative values represent instances where the *f_o_* of vowel following the fricative was lower than during the actual fricative production.

Relative *f_o_* measures shown in Figure 5, were also analysed.

The mean (*M*) and standard deviation of the mean (*SD*) of the relative values of *f_o_* for the three fricatives are shown in Table 4.

A simple linear model with relative *f_o_* as outcome variable and *group* as a binary categorical predictor, revealed a negative change (*slope*) from the CG to the SSD *group* that was significant (*slope* = −6.31, *SE* = 1.28, *p* < 0.001). Therefore, two regression models (one for each *group*—CG and SSD) with *f_o_* as outcome variable and *place* as a three-factor categorical predictor were used to further analyse the data, showing no significant effect of *place* on *f_o_* values (CG: *F*(2, 207) = 0.91, *p* = 0.403; SSD: *F*(2, 123) = 0.28, *p* = 0.756; omnibus tests).

**Figure 6:**
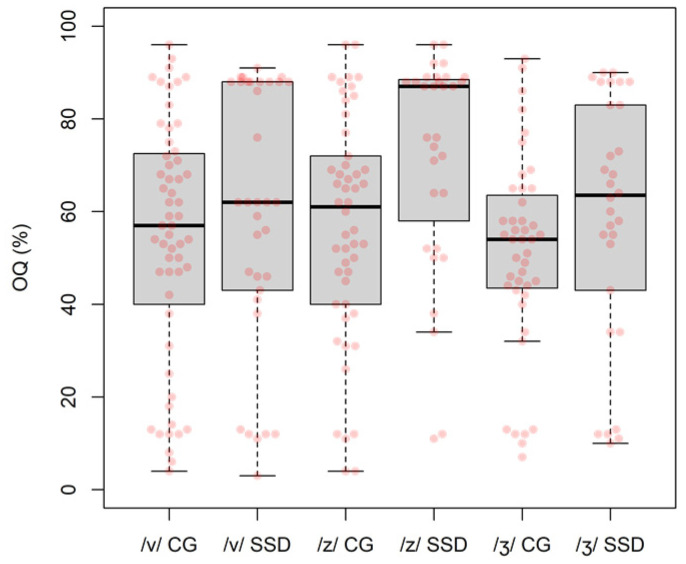
OQ data.

The OQ values, shown in Figure 6, were particularly difficult to estimate due to period-to-period instability of the EGG signal, and the fact the opening or closing peaks of the derivative of the EGG signal (used to calculate the OQ) were double or ill-defined [44].

The *mean* (*M*) and *standard deviation of the mean* (*SD*) of the OQ values for each *group* and *place* are shown in Table 5.

A linear regression framework was also used to model the relation between *OQ* (as outcome variable) and *group* (as a binary categorical predictor), showing a positive change (*slope*) from the CG to the SSD *group* that was significant (*slope* = 9.20, *SE* = 3.32, *p* = 0.006). Again, two regression models were developed with *OQ* as outcome variable and *place* as a three-factor categorical predictor, showing no significant effect of *place* on *OQ* values (CG: *F*(2, 146) = 0.73, *p* = 0.485; SSD: *F*(2, 92) = 2.33, *p* = 0.103; omnibus tests).

## 5. Discussion

Previous studies of children with SSD have shown that they have difficulties in producing and maintaining voicing in fricative sounds quite frequently [8,9,45,46], and that children with SSD have a lower AR than their peers without speech and language impairments [30,31,33,38,47]. Results such as these motivated the present study, with the central idea that oral and laryngeal articulation are related to the AR, aerodynamics and laryngeal/glottal configuration.

The use of aerodynamic data in the assessment of children’s speech production is under-reported in the literature. Thus, we used equipment and techniques previously tested in adult speakers to analyse the amplitude of the oral airflow, *f_o_* and OQ. Three measures, previously proposed by Pinho et al. [13], provided information about the oral airflow, the laryngeal configuration and vocal folds tension, which are important elements for the production and maintenance of voicing.

Children without SSD produced, on average (PPDN: *M* = 1.47, *SD* = 1.33), less than two phonological processes, whereas children with SSD produced more than four (PPDN: *M* = 4.62, *SD* = 2.02). Further analysis based on the picture naming task showed that all of PCC-R values for children in the CG (PCC-R: *M* = 98.45%, *SD* = 1.20%) were above the cut-off value (93.40% regardless of age) as previously reported in a study [48] for Brazilian Portuguese-speaking children; children with SSD all had PCC-R values (PCC-R: *M* = 79.69%, *SD* = 18.40%) that were lower than 93.40%.

During language development various factors can interfere with oral and laryngeal articulation control; anatomical growth, neurological and neuromuscular maturity, motor learning (that involves the planning and the motor programming of speech movements) as well as cognitive linguistic processing (including semantics, lexical and phonological access), all of which can impact motor control [36]. Some studies have shown that AR increases with age in typically developing children [30,34,35], a fact that may be due to the progressive maturity of the speech motor system [30,31,32,49].

The AR values of children with SSD (*M*_ARSS_ = 8.79 phones/s, *SD*_ARSS_ = 0.99 phones/s; *M*_ARLS_ = 10.50 phones/s, *SD*_ARLS_ = 1.36 phones/s) in our study were lower, on average, than those of children without SSD (*M*_ARSS_ = 9.28 phones/s, *SD*_ARSS_ = 1.02 phones/s; *M*_ARLS_ = 9.90 phones/s, *SD*_ARLS_ = 0.90 phones/s). Mean values are comparable to those previously reported [50,51] to be significantly different for the two groups (CG and SSD) in a large study involving more than 100 children with SSD (*M*_ARSS_ = 8.75 phones/s; *M*_ARLS_ = 9.90 phones/s) and 100 children without SSD (*M*_ARSS_ = 10.55 phones/s; *M*_ARLS_ = 11.35 phones/s), aged between 60 and 96 months.

The articulation rate values (ARSS and ARLS) observed in SSD children, were not significantly lower than those of children in the CG, but this could be one of the factors contributing to the different laryngeal articulation strategies observed in the two groups (as revealed by the relative *f_o_* values) and vocal efficiency (evidenced by OQ values). Speech and language interventions that control the articulation rate of children with SSD to be within an expected range for their age, could, as previously suggested by Boucher and Lamontagne [17], decrease oral pressure, which allows the maintenance of passive vocal fold vibration supported by a higher transglottal pressure without fully depending on the abduction of the vocal folds.

The percentage of weak-voiced fricative (RNWV) linear model showed a positive change from the CG to the SSD group that was not significant. Only an average of 1.3% (*SD* = 3.6%) of the voiced fricatives produced by children in the CG, during the standardised speech and language assessment, presented the phonological process of devoicing, but an average of 34.2% (*SD* = 30.0%) of those fricatives produced by children with SSD were devoiced. However, when one uses the relative oral airflow to classify voicing in fricatives produced in the current study, both the CG (*M* = 65.2%, *SD* = 18.3%) and the SSD group (*M* = 75.4%, *SD* = 13.8%) present a high number of weak-voiced fricatives. The SSD group was very heterogenous regarding voicing strategies, evidenced by the wide range of RNFD and RNWV values that is reflected on the high *SD* values shown above.

The RNFD values were calculated from phonetic transcription based on the auditory perception of speech by expert SLP, whereas the RNWV values were derived from the oral airflow signal. Portuguese fricatives have been shown [52] to be perceived as voiced/voiceless in various conditions with respect to the percentages of voicing maintenance (relative duration of voicing during fricative production) and adjacent vowels durations. Therefore, SLP’ assessments of voicing are the result of what is auditorily perceived as voiceless (in most of the children with SSD) based on the segmental durations of the fricative and adjacent phones. What we “see” based on the relative oral airflow measure is based on speech production data, which reflects the same weak-voicing strategy in the two groups and is also equivalent to what Portuguese-speaking adults produce [13]. The phonological processes used by children in the CG (very few fricatives were devoiced), were not reflected on the phonetic realisation of substantially less weak-voiced fricatives than for the SSD group.

The relative oral airflow (*flow*) linear model showed a positive change from the CG to the SSD group that was not significant, but a second regression model revealed that *place* had a statistically reliable effect on *flow*. A relative oral airflow of 70%, previously proposed [13] as a threshold between strong and weak -voicing, used to classify voicing in children, revealed that the probability of the SSD group using weak-voicing increased as the place of articulation moved backward, a behaviour that was similar to what had been previously observed for adult Portuguese speakers [13]. Place of articulation had the opposite effect for the group of children without SSD: Lower probability of weak voicing being produced for the most posterior fricatives. The pressure differential at the glottis needs to adjust to sustain voicing when the oral constriction moves backward (nearer the vocal folds), so the strategies adopted by children during the acquisition of voicing contrast are diverse.

There was a significant negative change from the CG relative *f_o_* values to those of the SSD group, and positive change in OQ from the CG to the SSD group was also significant. Lowering of laryngeal structures and vocal fold abduction that is used to maintain voicing during fricative production, results in lower *f_0_* values during fricative production than in the adjacent vowel [13]. This study’s results, revealing a significant negative change from the CG relative *f_o_* values to those of the SSD group, constitutes new evidence showing differences between children with and with SSD, in terms of their neuromuscular capacities and maturity of the motor speech system that control the two sound sources (oral and glottal). An increase in OQ value was further evidence that children with SSD have a less physically efficient voice, i.e., higher subglottal damping and weak sub- and supra-glottal acoustic excitation [13].

## 6. Conclusions

The aim of this study was to relate the measures inferred from the oral airflow and EGG signals to data routinely collected by SLP during speech and language assessments, in order to characterise the oral and laryngeal articulation strategies used by children with and without SSD to control voicing, as a function of place of articulation.

Articulation rate and relative oral airflow in children with and without SSD were not significantly different, but a statistically reliable effect of place on flow was found. Children with SSD used lower relative *f_o_* values than children without SSD (i.e., inadequate *f_o_* adjustments to maintain voicing), and the OQ values of children with SSD were higher than children without SSD, i.e., less efficient voicing. Place of articulation had heterogenous effects (among and amongst groups) on the strength of voicing.

One of the limitations of this study was the sample size due to large data losses, so future work should involve a larger number of children and closer monitoring of acoustic, oral airflow and EGG signal acquisition, avoiding problems with data analysis. Analysis of data on voiceless fricatives, and acoustic and auditory perception assessment of children with and without SSD should be included in future work.

Further studies are necessary in order to determine other factors that could be related to the voicing of fricative and stop consonants in children with SSD as it could help clinicians to improve treatment.

## Figures and Tables

**Table 1:** List of words with the voiced fricatives.

Portuguese	Phonetic Transcription	English
<Vidro>	/ˈvidru/	<Glass>
<Vela>	/ˈvɛlɐ/	<Candle>
<Zebra>	/ˈzebɾɐ/	<Zebra>
<Zero>	/ˈzɛru/	<Zero>
<Jogo>	/ˈʒoɡu/	<Game>
<Jota>	/ˈʒɔtɐ/	<J> (the letter <j>)

**Table 2:** Outcomes of an initial speech and language assessment and mean values of aerodynamic and EGG-based measures of the control group.

Child	Sex	Age (Months)	PPFD	PPTN	PPDN	PCC-R (%)	PDI	ARSS (Phones/s)	ARLS (Phones/s)	*M_flow_* (%)	*M_fo_* (%)	*M_OQ_* (%)	RNFD (%)	RNWV (%)
1	F	86	0	0	0	100	0.00	8.79	9.65	91.35	21.18	66.69	0.00	88.24
2	M	82	0	0	0	100	0.00	7.52	10.53	68.00	12.26	34.33	0.00	68.75
3	F	89	0	2	2	97.70	0.05	11.38	8.72	68.24	11.07	51.00	0.00	35.29
4	F	90	0	1	1	98.88	0.02	10.03	10.09	79.70	1.04	26.38	0.00	76.47
5	F	86	0	0	0	100	0.00	8.80	10.59	78.77	2.92	83.41	0.00	84.62
6	F	61	0	4	4	96.60	0.11	8.15	10.07	64.03	4.32	45.48	0.00	64.71
7	M	63	0	1	1	97.77	0.05	8.94	10.61	91.44	9.14	34.06	0.00	61.54
8	F	87	0	3	3	97.70	0.08	9.21	9.05	85.81	11.49	54.82	0.00	84.62
9	F	87	0	1	1	98.80	0.02	10.02	10.93	71.85	4.22	58.84	0.00	73.33
10	F	88	0	2	2	97.70	0.05	9.58	13.77	61.20	2.06	56.61	0.00	55.56
11	F	75	0	0	0	100	0.00	10.34	10.20	85.98	8.75	53.87	0.00	72.22
12	F	88	0	2	1	97.77	0.05	9.27	8.51	63.42	8.11	51.82	0.00	72.22
13	F	80	0	1	1	98.80	0.02	8.72	9.76	61.46	4.57	68.00	0.00	16.67
14	F	88	0	0	0	100	0.00	9.50	10.38	79.84	10.84	58.50	0.00	50.00
15	M	82	1	3	3	97.70	0.05	7.70	13.26	63.21	6.65	53.65	11.11	86.67
16	M	88	0	3	3	96.60	0.08	9.06	10.67	72.86	-2.87	70.17	0.00	61.11
17	M	89	1	3	3	97.70	0.08	10.74	10.90	77.58	17.77	77.24	11.11	57.14

**Table 3:** Outcomes of an initial speech and language assessment and mean values of aerodynamic and EGG-based measures of children with SSD.

Child	Sex	Age (Months)	PPFD	PPTN	PPDN	PCC-R (%)	PDI	ARSS (Phones/s)	ARLS (Phones/s)	*M_flow_* (%)	*M_fo_* (%)	*M_OQ_* (%)	RNFD (%)	RNWV (%)
1	M	88	8	39	6	57.77	1.14	8.51	8.97	93.82	−15.55	71.41	88.89	60.00
2	F	61	0	20	4	90.0	0.26	8.14	8.31	80.81	3.22	74.58	0.00	87.50
3	M	79	1	10	4	88.88	0.29	8.60	9.91	74.92	−6.97	54.87	11.11	75.00
4	M	60	5	12	3	86.66	0.35	8.56	9.08	87.62	5.50	85.83	55.56	88.24
5	M	82	5	19	4	80.00	0.5	10.08	10.84	66.50	−1.21	66.72	55.56	69.23
6	M	68	2	9	5	90.00	0.26	9.12	9.24	85.84	−6.61	37.70	22.22	71.43
7	M	68	7	57	9	33.30	1.58	8.14	9.64	52.06	−1.97	63.00	77.78	53.85
8	M	80	2	8	4	90.80	0.24	6.80	9.75	80.86	8.71	62.00	22.22	55.56
9	M	92	0	1	1	98.89	0.03	7.90	9.99	87.95	25.19	71.67	0.00	72.22
10	M	64	1	35	6	61.11	1.02	9.22	10.20	79.52	14.53	50.00	11.11	100
11	M	87	3	6	4	93.33	0.17	8.77	11.49	70.92	−10.17	63.27	33.33	93.75
12	M	63	0	13	7	86.66	0.35	10.49	11.15	56.45	−6.16	54.49	0.00	82.35
13	M	80	6	21	3	77.70	0.60	9.90	10.10	77.61	0.00	78.20	66.67	71.43

**Table 4:** Mean (*M*), standard deviation of the mean (*SD*) and number of samples (n) of relative *f_o_* values for the three places of articulation: /v/—labiodental; /z/—alveolar; /ʒ/—postalveolar.

Group/Place	/v/			/z/			/ʒ/		
	*M* (%)	*SD* (%)	n	*M* (%)	*SD* (%)	n	*M* (%)	*SD* (%)	n
SSD	2.0	14.1	45	−0.3	14.7	41	0.3	15.1	40
CG	8.2	9.0	64	6.7	9.6	71	6.3	8.1	75

**Table 5:** Mean (*M*), standard deviation of the mean (*SD*) and number of samples (n) of OQ values for the three places of articulation: /v/—labiodental; /z/—alveolar; /ʒ/—postalveolar.

Group/Place	/v/			/z/			/ʒ/		
	*M* (%)	*SD* (%)	n	*M* (%)	*SD* (%)	n	*M* (%)	*SD* (%)	n
SSD	59.6	28.7	34	71.9	23.7	31	59.0	26.8	30
CG	54.2	26.1	56	57.1	24.4	50	51.0	21.3	43

## Data Availability

Data can be downloaded at: https://www.mdpi.com/article/10.3390/children9050649/s1, WertznerPaganJesus2022_data.xlsx.

## Supplementary Materials

The following supporting information can be downloaded at: https://www.mdpi.com/article/10.3390/children9050649/s1, WertznerPaganJesus2022_data.xlsx.
